# Is It Really a Failure? A Survey About Foster Animal Adoption

**DOI:** 10.3390/ani14233498

**Published:** 2024-12-04

**Authors:** Laura A. Reese

**Affiliations:** Michigan State University, East Lansing, MI 48824, USA; reesela@msu.edu

**Keywords:** animal foster care, foster adoption, animal sheltering

## Abstract

The widespread use of the term “foster fail” in animal rescue suggests that it happens often, but no research has explored the prevalence of volunteers adopting their foster animals or whether the phenomenon is really a “failure”. This survey-based study focused on the following questions: 1. How common are foster fails among volunteers on shelter and rescue lists and why do they occur? 2. What types of volunteers are most likely to adopt their foster animals? 3. Do different attachment styles to pets affect foster adoption? 4. Is the adoption of foster animals a way to deal with the potential grief of letting them go to adoption? 5. What are the impacts of foster fails on animal shelters in terms of longevity of volunteers and satisfaction with the volunteer experience? Data were collected through surveys of foster volunteers. Two nonprofit organizations, the Pedigree Foundation and Shelter Animals Count, distributed information about the survey and shelter directors distributed the survey link to their population of foster volunteers. Nine hundred and forty-seven individuals responded. To address the research questions, frequency, correlation, and regression analyses were employed. A total of 38% of volunteers had not adopted a foster in the past ten years, and another 38% had adopted one or two; 90 (11%) and 103 (13%) had adopted three to four or more than four, respectively. Volunteers that had significantly higher numbers of foster fails were those that were older (r = 0.22, *p* < 0.001), retired (chi-squared = 9.05, *p* = 0.029), lower on educational attainment (r = −0.13, *p* < 0.001), female with their own cats (r = 0.16, *p* < 0.001), and part of a fostering family (r = 0.08, *p* = 0.043). Volunteers that expressed higher levels of both people-substituting (r = 0.16, *p* = 0.003) and general (r = 0.13, *p* = 0.017) attachment to their fosters were more likely to adopt them, as were those that more frequently fostered animals with special medical or behavioral needs (r = 0.25, *p* < 0.001). Volunteers that had longer tenures (r = 0.43, *p* < 0.001), fostered more frequently (r = 0.24, *p* < 0.001), and reported greater resilience (r = 0.10, *p* = 0.009) had adopted significantly more animals. Finally, there was a significant and positive relationship between satisfaction with fostering and adopting more foster animals (r = 0.16, *p* < 0.001). The findings indicated that instead of being a “failure,” foster adoptions can be a positive force for the animal in question, their adopters, and shelters and rescues because they have more resilient, satisfied, and active volunteers.

## Simple Summary

The widespread use of the term “foster fail” in animal rescue suggests that it happens often, but no research has explored the prevalence of volunteers adopting their foster animals or whether the phenomenon is really a “failure”. This survey-based study of 947 foster volunteers focused on the following questions: 1. How common are foster fails among volunteers on shelter and rescue lists and why do they occur? 2. What types of volunteers are most likely to adopt their foster animals? 3. Do different attachment styles to pets affect foster adoption? 4. Is the adoption of foster animals a way to deal with the potential grief of letting them go to adoption? 5. What are the impacts of foster fails on animal shelters in terms of longevity of volunteers and satisfaction with the volunteer experience? The findings suggest that 38% of volunteers had not adopted a foster in the past ten years and another 38% had adopted one or two. Volunteers most likely to adopt their foster animals were female with their own cats, older and retired, and those that reported greater people-substituting attachment to their fosters. Foster fails were not associated with less frequent fostering; volunteers with more foster fails reported significantly higher resilience scores and were more satisfied with their fostering experience.

## 1. Introduction

Fostering is critical to the animal welfare mission of shelters and rescues. Euthanasia due to overcrowding is common; estimates of animals euthanized in shelters annually ranged from 415,000 to 850,000 in 2023 [1,2]. Getting animals out of the shelter and into foster homes addresses capacity limitations. But there are other benefits of placing animals into foster. Shelters are a stressful environment, risking animal well-being and adoptability. Animals in shelters often hear and see other animals but typically do not get to interact with them, get limited exercise, lack freedom of movement, may have few opportunities to interact with people, and cannot make their own decisions [3,4].

Among animal shelter professionals and foster volunteers, the notion of a “foster fail” is well-understood. It refers to when a volunteer adopts their foster animal and there have been calls in professional arenas to rename it so that it represents a “success” [5,6,7]. If volunteers regularly adopt their fosters, then there is a risk to animal shelters that they may not continue their very needed service, particularly if the adopted animal does not like other pets. Further, shelters and rescues put time and effort into recruiting and training foster volunteers, so from a resource standpoint, retentionis important. But, how common are foster fails? The widespread use of the term in animal rescue would suggest that it happens often, but no research has explored the prevalence of volunteers adopting their foster animals or whether the phenomenon is really a “failure”.

The dearth of studies on foster fails makes the discussion of previous work difficult. Yet, there are strands of research that set the stage for examining the adoption of one’s foster animals. First, there are a number of benefits of foster programs. Shelters and rescues that have more foster volunteers, a foster coordinator to manage the program, and more animals in foster homes have been found to have lower euthanasia rates than those with weaker or no foster programs [8,9,10]. Being in a foster home can lead to quicker adoptions because animals can avoid the stress of the shelter, and behavioral or medical barriers can be addressed [11,12]. Fostering also reduces the chances that an animal will be returned to the shelter because volunteers will have more information to pass on to potential adopters, particularly regarding interactions with other animals and household members such as children, allowing for a better match between pet and adoptive home [9,13].

These benefits rely on volunteers willing to take animals into their homes, potentially becoming attached. The limited research on attachment to foster animals suggests that volunteers become as attached to them as do people to their own pets [14,15]. This attachment can be a dual edged sword. On the one hand, there appears to be a variety of benefits; volunteers who foster dogs report higher levels of physical activity [16,17], and animal foster volunteers indicate that their charges provide companionship and emotional support [15]. On the other hand, strong attachment to pets has been found to be related to depression [18], as well as an increased risk of a variety of other diseases [19,20]. Emotional attachment to pets can cause intense grief when the animal dies at a level similar to the loss of a human family member [21,22]. Research on families that provide foster care for human children has shown that concerns about the child leaving are highly stressful, causing “anticipatory grief” to the extent that individuals may stop fostering altogether [23,24]. Strong attachments to foster animals could lead to foster fails in an effort to mitigate feelings of anticipatory grief.

This survey-based study focuses on the following questions:

1. How common are foster fails among volunteers on shelter and rescue lists and why do they occur?
2. What types of volunteers are most likely to adopt their foster animals?
3. Do different attachment styles to pets affect foster adoption? It is expected that volunteers that are more strongly attached to their fosters will be more likely to adopt them.
4. Is the adoption of foster animals a way to deal with the potential grief of letting them go to adoption? It is logical that volunteers who anticipate grief when their foster animals leave the home are more likely to adopt them.
5. What are the impacts of foster fails on animal shelters in terms of longevity of volunteers and satisfaction with the volunteer experience?

## 2. Methods

### 2.1. Participants and Survey

The data for this study were collected through electronic self-administered surveys of foster volunteers, recruited between March and September of 2023. Two nonprofit organizations, the Pedigree Foundation (1350 members) and Shelter Animals Count (SAC) (11,000 subscribers), distributed information about the survey to their lists of members, shelters, and rescues via listservs and online newsletters. The directors of the member organizations were asked to distribute the survey link to their total population of foster volunteers, which could include both active and inactive fosters. Because of the administration method, it was not possible to know how the participating shelters and rescues kept their volunteer lists. Most importantly, whether the lists definitely included both active and inactive fosters is not known. It is likely that inactive foster volunteers are underrepresented in the sample because they were either not included on the distribution list or were less likely to respond to the survey. Past research of other shelters and rescues has indicated that the list of potential volunteers is larger than that of active volunteers at most shelters, yet many organizations keep inactive volunteers on their lists, suggesting that inactive foster volunteers were sent the survey link for this project [25].

A consent form began the survey, and respondents were asked to indicate whether they agreed to participate. A “yes” response allowed them to continue on to the survey questions. The study received exempt approval status from the Institutional Review Board at Michigan State University, Study 00008888 for the Pedigree Foundation and Study 00009612 for Shelter Animals Count. The Foster Care Survey was administered using Qualtrics, and all responses were recorded confidentially. This was primarily a bespoke survey due to the dearth of research exploring the experiences of animal foster care providers. However, a number of the scales were drawn from other research and represent validated measures (see discussion of measures and indicators). The survey was not focused specifically on foster adoption but included questions about a variety of aspects of the foster volunteer experience: other types of service within the shelter; motivations for providing foster care; breaks in foster service; types of animals fostered; self-care practices; and general views about fostering. Only those questions related to foster fails, volunteer characteristics, feelings of grief, resilience, and satisfaction are employed here with all measures and validation as described below.

### 2.2. Measures and Indicators

Individual questions on the survey were combined into indexes based on validation in previous research and to avoid multicollinearity in the regression analysis. Volunteer characteristics were used individually and combined using exploratory factor analysis. Attachment to foster pets, feelings of grief, resilience, and satisfaction were combined into indexes based on confirmatory factor analysis (all presented in Table 1).

Volunteer Characteristics: To identify what types of volunteers are most likely to adopt their fosters, the correlation analysis employed individual variables such as age, education. and gender but for the purposes of multiple regression, volunteer traits were combined to form indexes to reduce multicollinearity. Four conceptually different aspects of volunteer traits and circumstances emerged from the factor analysis; fosters that are older and retired, female volunteers with their own cats, family fosters with two adults and children in the home, and home-owning volunteers with higher education.

Attachment to Pets: The Lexington Attachment to Pets Scale (LAPS) was employed in this research because it is one of the most widely cited pet attachment questionnaires and is among the most frequently validated [26,27,28]. To minimize survey length, only those questions representing the general attachment and people-substituting subscales were employed. It should be noted that there have been criticisms of the validity of the LAPS including concerns about the inclusion of the questions related to animal welfare, the use of reverse-scored questions, and the fact that it does not consider that bondedness may vary by species of animal [29]. Two of these concerns were addressed here by removing the animal welfare questions and using all positively scored responses.

Nature of Foster Service: The survey included individual questions asking how long and with what frequency respondents have fostered animals. Animals are in foster for different reasons, which may affect foster adoptions. The survey included two questions on the frequency of fostering animals with special medical or behavioral needs and these were combined into a single index.

Foster Volunteer Grief: The foster parent grief scale developed by Theut and colleagues was used to measure grief [24,30,31]. Confirmatory factor analysis indicated that all of the grief questions loaded on the same factor.

Resilience: Resilience has been defined as the ability to cope with stress and recover quickly when it occurs [32]. Emotional resilience of human foster care providers has been found to be associated with stability in placements [33], and if applied to animal foster volunteers, might be related to tenure and satisfaction. The survey included questions drawn from the Brief Resilience Scale developed by Smith and colleagues [34]; confirmatory factor analysis showed the presence of a single resilience index.

Satisfaction: Finally, five questions were used to indicate satisfaction with fostering generally and with specific organizations based on previous research [14,35].

### 2.3. Analysis

To address the research questions, frequency, correlation, and regression analyses were employed. For nominal level variables such as gender, Pearson chi-squared statistics were calculated to assess correlation. For ordinal or interval level variables Pearson correlations are presented. For correlations with ordinal-level variables, Spearman’s correlations and Kendall’s tau-b were also examined, and the results did not differ from those of the Pearson correlations. All analyses were conducted using IBM SPSS Statistics 27.

## 3. Results

Nine hundred and forty-seven individuals responded to the survey. If volunteers worked for both a Pedigree and a SAC organization, they may have received the survey link more than once. IP addresses were checked for duplicates, and one survey response was removed from the dataset because of duplication, leaving a total of 946 responses. Because of the survey length, there appeared to be some survey fatigue creating missing data. The missing data were accepted, and pairwise deletion was employed; all frequency tables indicate the number of responses for each question.

Responding foster volunteers were overwhelmingly female (92%), between 26 and 65, and were quite well-educated: 38% had a bachelor’s degree and 32% had graduate or professional degrees (Table 2). Seventy-six percent of responding foster volunteers did not have any children in their home but lived with another adult, only 28% lived alone, and 79% owned a single-family home.

Table 3 shows the length of service and frequency of fostering for the respondents. The plurality (283 or 35%) had been fostering for one to three years; another 23% were new volunteers fostering for less than a year. On the other hand, 42% of the respondents had been fostering for four or more years. Forty-one percent of the respondents foster several times a year, while 37% have foster animals in their homes most of the time. It appears that the sample thus includes both new and long-term foster volunteers and favors those that have animals at least occasionally or frequently. Table 3 also provides frequencies for the number of foster fails. Thirty-eight percent of volunteers had not adopted a foster in the past ten years, and another 38% had adopted one or two; 90 (11%) and 103 (13%) had adopted three to four or more than four, respectively.

Reported reasons for adopting one’s foster are shown in Table 4 in the order of the most common based on mean scores on a five-point scale. The most frequent reason for adopting a foster animal is because the volunteer fell in love with them and could not let them go to adoption; 407 (58%) of the respondents agreed or strongly agreed with this statement (mean = 3.54). The next most common reasons for adoption were that the animals appeared so happy in the foster home that the volunteer did not have the heart to move them (mean = 2.94) or because they had behavioral or medical issues that would have made adoption difficult (mean = 2.88).

The data in Table 5 show the relationships between the number of fosters adopted; volunteer traits; attachment style; nature of the foster service; and emotions such as grief, resilience, and satisfaction. Volunteers that had significantly higher numbers of foster fails tended to be older (r = 0.22, *p* < 0.001), retired (chi-squared = 9.05, *p* = 0.029), lower on educational attainment (r = −0.13, *p* < 0.001), female with their own cats (r = 0.16, *p* < 0.001), and part of a fostering family (r = 0.08, *p* = 0.043). Volunteers that expressed higher levels of both people-substituting (r = 0.16, *p* = 0.003) and general (r = 0.13, *p* = 0.017) attachment to their fosters were more likely to adopt them, as were those that more frequently fostered animals with special medical or behavioral needs (r = 0.25, *p* < 0.001). Volunteers that had longer tenures (r = 0.43, *p* < 0.001), foster more frequently (r = 0.24, *p* < 0.001), and reported higher scores on the resilience scale (r = 0.10, *p* = 0.009) adopted significantly more animals. Finally, there was a significant and positive relationship between satisfaction with fostering and adopting more foster animals (r = 0.16, *p* < 0.001). Grief was not significantly correlated with adopting a foster animal.

To assess the relative importance of the variables significantly correlated with foster fails in the bivariate analysis, an ordinary least squares regression was run with number of fosters adopted as the dependent variable (only the volunteer trait indexes were used to avoid multicollinearity). The variables in the model accounted for 17% of the variation in numbers of fosters adopted, with the length of foster service being the best predictor based on the beta values (Table 6). Higher levels of people-substituting attachment and resilience, as well as being female with one’s own cats, also remained significantly correlated to foster fails in multiple regression. The variation inflation factors (VIF) that indicate potential multicollinearity were all below the cut point of 2.5, which indicates collinearity.

The best fitting regression equation is presented in Table 7, accounting for 18% of the variance in foster fails. Those adopting more foster animals had been fostering longer, reported more people-substituting attachment, were higher on the resilience scale, and were female with cats. Again, length of foster service was the best predictor of the number of animals adopted based on the beta values.

To provide another perspective on these findings, the number of fosters adopted, resilience, length of service, and satisfaction with the fostering experience were entered into a factor analysis (Table 8). All of these variables appeared to be part of a single underlying concept that could be considered to represent a complex of beneficial aspects of providing foster care.

## 4. Discussion

The discussion of the results is organized around the five research questions posed at the beginning of the paper.

### 4.1. How Common Are Foster Fails Among Volunteers on Shelter and Rescue Lists and Why Do They Happen?

Thirty-eight percent of foster volunteers responding to the survey had not adopted a foster in the past ten years, and another 38% had adopted only one or two. In another question, 21% agreed or strongly agreed that they were fostering with the intention of potentially adopting the animal. On the one hand, it appears that many foster adoptions are the unintended result of strongly bonding with a particular pet. However, it might behoove shelters and rescues to identify volunteers that are fostering with the intention of finding a pet and those that are fostering for the longer term. This information would allow organizations to better direct and organize resources to support the different needs of volunteers. The next most common reasons for adopting fosters lie more with the nature of the particular animal, either because it appeared to be happy in the foster home or because of behavioral or medical issues that would have made adoption difficult. As noted previously, it is possible that the data are measuring foster fails only among volunteers currently fostering. However, shelters were asked to distribute the survey link to their entire population of fosters, lists of which could include those that were currently inactive, potentially because they had previously adopted their foster animals.

### 4.2. What Types of Volunteers Are Most Likely to Adopt Their Foster Animals?

Based on the results of the multiple regressions, the volunteers most likely to adopt their foster animals are those that are female with their own cats and that are older and retired. This is consistent with other research finding a correlation between being female and choosing to foster cats, and that individuals with their own cats are more likely to foster cats than dogs [36]. Resilience is also significantly and positively correlated with the number of foster fails. Because this study is cross-sectional, it is possible that more resilient volunteers are more likely to adopt their fosters.

### 4.3. Do Different Attachment Styles to Pets Affect Foster Adoption?

Volunteers who report higher people-substituting attachments to animals are more likely to adopt their fosters. Specifically, those that express that their relationships to animals are closer and more fulfilling than relationships to other humans are more likely to become attached to their foster animals and adopt them.

### 4.4. Is the Adoption of Foster Animals a Way to Deal with the Potential Grief of Letting Them Go to Adoption?

Foster animal adoption and expressions of grief when animals leave the home are not significantly correlated. In short, even those volunteers that have more feelings of sadness and loss when an animal is adopted were not more likely to keep it.

### 4.5. What Are the Impacts of Foster Fails on Animal Shelters in Terms of Longevity of Volunteers and Satisfaction with the Volunteer Experience?

One of the concerns with foster failures is that volunteers may end up with so many animals that they decide to stop fostering. It could also be the case that they might worry about their ability to let future fosters go to adoption. This concern appears unwarranted, and indeed those that have adopted more fosters volunteer more frequently and have longer tenures. The survey was cross-sectional, however, so it is not possible to determine the order of causation with certainty. Thus, fostering for longer periods of time and with more frequency may lead to adoptions simply because the volunteer has come in contact with more animals that they might become attached to. On the other hand, it does not appear that foster fails are associated with less frequent fostering or with volunteers deciding to end their service at least among those that received the survey link. It is also important to note that volunteers with more foster fails are significantly more satisfied with both the enterprise of fostering generally and with their current organization specifically.

## 5. Limitations

This study has a number of important limitations. The most significant is that the nature of the lists of fosters used by the participating organizations is unknown. While shelter and rescue directors were asked to send the survey link to their population of volunteers as opposed to just those that were active, procedures for purging lists of inactive volunteers are unknown. This means that volunteers who have adopted fosters and stopped fostering as a result may or may not have received the survey. Thus, the responses likely overrepresent active foster volunteers. The sample was relatively balanced between volunteers with different tenures (new and longer term) and those that foster several times a year and most of the time are overrepresented. This suggests that many volunteers who have adopted their foster animals were still included in the results. To examine this more carefully, future surveys could be conducted with a larger public sample that might better represent the attitudes of foster volunteers who adopted animals and then stopped their service. Another potential avenue for research would be to examine individual shelter and rescue data to identify how many and what types of volunteers ended their service upon adoption of a foster animal.

Another potential limitation is the use of the LAPS to measure attachment to foster pets. The modifications to the questions used here (all positively coded and animal welfare questions excluded) reduce concerns about the validity of the measure, but it still may not have been equally applicable to foster pets of different species. Additionally, there was evidence of survey fatigue. The volunteers that stopped answering the survey at some point may be different than those that saw it to completion. This likely creates a bias toward more committed volunteers or those with stronger opinions.

The survey and resulting data are cross-sectional, and thus it is not possible to establish causal sequencing. For example, more resilient volunteers may be more likely to adopt their foster animals as opposed to adoption enhancing resilience. However, the large body of literature suggesting mental health benefits of pets supports the latter. Similarly, more satisfied volunteers may be more likely to adopt their foster animals.

The survey did not ask what species of animal foster volunteers adopted. The representation of cat and dog fosters among the respondents was very similar, and thus there appeared to be no response bias in type of animal fostered. However, the significant correlation between women with their own cats and foster fails suggests that future research should look more closely at the relationships between type of animals fostered, species of animal adopted, and gender.

While the IP addresses of respondents were checked for duplicates, it was not possible to tell if multiple people in the same household answered the survey using different addresses.

More generally, the respondents were drawn only from shelters and rescues in the US and thus may not be representative of those in other countries. Another potential concern about representativeness of the sample is the large number of female versus male respondents. Studies of pet owners generally have found them more likely to be female, between 30 and 39, and with children in the home [37]. Recent surveys of animal foster volunteers have had similar sample profiles, with respondents being predominantly female, white, and with other pets in the home [14,38,39]. Respondents to this survey appear representative of foster volunteers generally; however, future research could strive to identify equal numbers of male and female fosters to examine both attachment styles and the tendency to adopt.

## 6. Conclusions

The findings indicate that while roughly 60% of volunteers have adopted a foster animal, only half of those have kept more than one or two animals. Further, volunteers who have adopted their foster animals are more satisfied with their fostering experiences and with their shelter or rescues, potentially leading to continued and more frequent service. In short instead of being a “failure”, foster adoptions could be a positive force for the animal in question, their adopters, and shelters and rescues because they have more resilient, satisfied, and active volunteers.

## Figures and Tables

**Table 1:** Factor analysis results, and indexes used in further analyses.

	Factor Loading
**Older/retired**	
Age	0.84
Not working	0.84
**Family fosters**	
Own dogs	0.53
Share home	0.75
Number of children	0.70
**Higher SES**	
Education	0.74
Own home	0.74
**Female with cats**	
Female	0.56
Own cats	0.56
**General attachment**	
I play with my fosters quite often	0.73
Fostering adds to my happiness	0.80
My fosters and I generally have close relationships.	0.80
I consider my fosters to be great companions	0.84
I often talk to other people about my fosters	0.64
Interacting with my fosters helps me stay healthy	0.67
**People-substituting attachment**	
My fosters know when I am feeling bad	0.69
I love my fosters because they never judge me	0.71
Quite often I confide in my fosters	0.76
My feelings toward people are affected by the way they react to my fosters	0.65
My foster animals mean more to me than many of my friends	0.83
My fosters understand me	0.86
My fosters are more loyal to me than most people in my life	0.83
I consider my fosters to be my friends	0.80
**Grief**	
Daydream about life with foster	0.59
More irritable since foster left	0.76
Preoccupied with thoughts about foster	0.80
Very much miss foster	0.63
Feel very much alone since foster left	0.75
Hard to concentrate on work	0.72
Have periods of tearfulness when I think about foster	0.77
Need to talk to others about the loss I feel	0.74
No child/animal will ever take the place of foster	0.61
**Resilience**	
I am able to adapt to change	0.70
I can deal with whatever comes	0.75
I try to see the humorous side of problems	0.58
Coping with stress can strengthen me	0.62
I tend to bounce back after illness or hardship	0.71
I can achieve goals despite obstacles	0.76
I can stay focused under pressure	0.71
I am not easily discouraged by failure	0.73
I think of myself as a strong person	0.69
I can handle unpleasant feelings	0.72
**Satisfaction with fostering**	
Can’t see stopping fostering	0.79
Pleasures outweigh disadvantages	0.85
Makes feel good and raises self-esteem	0.64
**Satisfaction with organization**	
Like volunteering with organization	0.94
Satisfied with volunteer experience	0.94

N = 946.

**Table 2:** Frequencies and characteristics of respondents.

	Full Sample N (%) *	Pedigree N (%)	SAC N (%)
Gender			
Female	660 (92)	394 (93)	281 (92)
Male	40 (6)	23 (5)	19 (6)
Gender variant/non-conforming	6 (1)	3 (1)	3 (1)
Not listed	1 (0.1)	1 (0.2)	0 (0)
Refused	8 (1)	4 (1)	4 (1)
Age			
18–25	28 (4)	16 (4)	4
26–45	298 (42)	180 (43)	41
46–65	286 (40)	176 (42)	38
66 and over	101 (14)	52 (12)	17
Education			
Some high school, no diploma	2 (0.3)	1 (0.2)	2 (1)
High school graduate	33 (5)	24 (6)	10 (3)
Some college	77 (11)	44 (10)	35 (12)
Trade/technical/vocational training	36 (5)	18 (4)	18 (6)
Associate degree	65 (9)	35 (8)	31 (10)
Bachelor’s degree	271 (38)	171 (40)	108 (35)
Master’s degree	172 (24)	102 (24)	73 (24)
Professional degree	20 (3)	11 (3)	9 (3)
Doctorate degree	38 (5)	20 (5)	19 (6)
Housing			
Own single-family home	562 (79)	338 (80)	236 (77)
Own in a multi-family building	29 (4)	19 (5)	10 (3)
Rent single-family home	53 (7)	30 (7)	26 (9)
Rent in a multi-family building	59 (8)	35 (8)	24 (8)
Live with family/friends	11 (2)	3 (1)	10 (3)
Children < 18 in the home			
0	538 (76)	337 (80)	215 (70)
1–2	146 (21)	71 (17)	78 (26)
3–4	26 (4)	15 (4)	11 (4)
More than 5	3 (0.4)	1 (0.2)	2 (1)
Other adults in home			
Spouse/romantic partner	450 (64)	268 (64)	191 (63)
Other family member	51 (7)	26 (6)	28 (9)
Roommate	9 (1)	9 (2)	3 (1)
Live alone	198 (21)	119 (28)	81 (27)

* The numbers of respondents to the Pedigree and Shelter Animals Count (SAC) surveys do not add up to the numbers of total respondents because duplicates were removed when the sets were combined.

**Table 3:** Frequencies: length and frequency of foster service.

Tenure	N (%)
Less than a year	188 (23)
1–3 years	283 (35)
4–7 years	145 (18)
7–10 years	77 (9)
More than 10 years	122 (15)
**Frequency of Fostering**	
One time	72 (9)
Occasional/several times a year	337 (41)
Seasonal/kitten season or when not working	105 (13)
Frequent/have fosters most of the time	301 (37)
**Number of Foster Fails Over Past 10 Years**	
None	312 (38)
1–2	312 (38)
3–4	90 (11)
More than 4	103 (13)

**Table 4:** Frequencies: stated reasons for adopting foster animals.

	Strongly Disagree N (%)	Disagree N (%)	Neither Agree Nor Disagree N (%)	Agree N (%)	Strongly Agree N (%)	Mean
I just fell in love with my foster and could not let it go	124 (18)	55 (8)	117 (17)	133 (19)	274 (39)	3.54
The foster seemed so content I didn’t have the heart to move him/her	186 (27)	80 (12)	154 (22)	143 (21)	133 (19)	2.94
My foster had challenges that would have made it very hard to find an adoptive home	250 (36)	53 (8)	100 (14)	134 (19)	167 (24)	2.88
My children fell in love with the foster	311 (45)	16 (2)	207 (30)	58 (8)	96 (14)	2.44
I was fostering with the intention of adopting if the fit was right	347 (50)	81 (12)	119 (17)	70 (10)	78 (11)	2.21

**Table 5:** Pearson correlations and chi-squares: independent variables and the number of fosters adopted over the past ten years.

	Number of Fosters Adopted: Pearson Correlations and Chi-Squares
Volunteer Traits	
Age	0.22 **
Education	−0.13 **
Gender	6.36 ^
Children under 18	−0.02
Own home	18.45 ^
Work at home	0.24 ^
Work outside home	3.02 ^
Hybrid	3.51 ^
Retired	9.05 ^*
Indexes	
Older/retired	0.17 **
Family fosters	0.08 *
Higher SES	−0.06
Female with cats	0.16 **
Attachment Style	
General	0.13 *
People-substituting	0.16 **
Nature of Foster Service	
Tenure	0.43 **
Frequency	0.24 **
Special needs	0.25 **
Foster Emotions	
Grief	−0.02
Satisfaction with fostering	0.16 **
Satisfaction with organization	0.09 *
Resilience	0.10 **

^ = chi-squared; * = significant at 0.05; ** significant at 0.01.

**Table 6:** Full regression results: dependent variable = number of fosters adopted over the past ten years.

	B	Standard Error	Beta	t	Sig	VIF
Tenure	0.22	0.06	0.29	4.08	0.00	1.63
Frequency	0.06	0.06	0.06	0.89	0.37	1.35
Older/retired	0.04	0.06	0.04	0.71	0.48	1.11
Special needs	−0.02	0.07	−0.03	−0.35	0.73	1.60
People-substituting attachment	0.19	0.07	0.19	2.83	0.01	1.49
General attachment	−0.02	0.07	−0.02	−0.28	0.78	1.39
Female with cats	0.11	0.06	0.12	2.05	0.04	1.09
Family fosters	0.06	0.06	0.07	1.15	0.25	1.13
Education	−0.04	0.04	−0.06	−0.99	0.32	1.19
Resilience	0.15	0.05	0.16	2.76	0.01	1.05
Constant	1.43	0.30		4.84	0.00	
Adjusted R^2^ = 0.17						

**Table 7:** Reduced regression results: dependent variable = number of fosters adopted over the past ten years.

	B	Standard Error	Beta	t	Sig	VIF
Tenure	0.23	0.04	0.30	5.53	0.00	1.04
Female with cats	0.13	0.05	0.14	2.51	0.01	1.05
People-substituting attachment	0.19	0.05	0.20	3.64	0.00	1.01
Resilience	0.16	0.05	0.17	3.17	0.00	1.01
Constant	1.37	0.11		12.18	0.00	
Adjusted R^2^ = 0.18						

**Table 8:** Factor analysis results: benefits of foster fails.

	Factor Loading
Benefit System	
Tenure	0.76
Total fosters adopted	0.78
Resilience	0.36
Satisfaction with fostering	0.50

## Data Availability

Data are available upon request.

## References

[1] Best Friends Animal Society National Animal Shelter Statistics Dashboard. Best Friends Animal Society..

[2] (2023). Shelter Animals Count. Data summary. 2023 Statistics—Shelter Animals Count.

[3] Gunter L.M., Feuerbacher E.N., Gilchrist R.J., Wynne C.D. (2019). Evaluating the effects of a temporary fostering program on shelter dog welfare. PeerJ.

[4] Thielke L.E., Udell M.A. (2019). Characterizing human–dog attachment relationships in foster and shelter environments as a potential mechanism for achieving mutual wellbeing and success. Animals.

[5] Foster Fail: Why Foster Failures Are Actually a Good Thing. https://adoptapet.com.

[6] When Fostering Leads to Forever: Embracing the “Foster Fail”. https://rescuehund.com.

[7] What Is a Foster Fail?. Vet-Reviewed Facts & FAQ—Dogster..

[8] Patronek G.J., Crowe A. (2018). Factors associated with high live release for dogs at a large, open-admission, municipal shelter. Animals.

[9] Reese L.A. (2021). Shelter and rescue programs associated with higher live release and lower return rates for dogs. Anim. Welf..

[10] Rodriguez J.R., Davis J., Hill S., Wolf P.J., Hawes S.M., Morris K.N. (2022). Trends in intake and outcome data from US animal shelters from 2016 to 2020. Front. Vet. Sci..

[11] Taraciuk A.C., Leite L.O., Polo G., Garcia R.D.C.M. (2020). An overview of animal foster homes in Brazil. Arch. Vet. Sci..

[12] Thumpkin E., Paterson M.B., Morton J.M., Pachana N.A. (2022). Adoption can be a risky business: Risk factors predictive of dogs adopted from RSPCA Queensland being returned. Animals.

[13] Mohan-Gibbons H., Weiss E., Garrison L., Allison M. (2014). Evaluation of a novel dog adoption program in two US communities. PLoS ONE.

[14] Reese L.A., Jacobs J., Seelenbinder B., Stedhouwer T., Velychko N., Wathen L. (2023). The emotional aspects of dog fostering: Both ends of the leash. Anthrozoös.

[15] Powell L., Ackerman R., Reinhard C.L., Serpell J., Watson B. (2024). A prospective study of mental wellbeing, quality of life, human-animal attachment, and grief among foster caregivers at animal shelters. PLoS ONE.

[16] Potter K., Teng J.E., Masteller B., Rajala C., Balzer L.B. (2019). Examining how dog ‘acquisition’ affects physical activity and psychosocial well-being: Findings from the buddystudy pilot trial. Animals.

[17] Ortmeyer H.K., Robey L.C. (2019). Companion dog foster caregiver program for older veterans at the VA Maryland Health Care System: A feasibility study. Int. J. Environ. Res. Public Health.

[18] Miltiades H., Shearer J. (2011). Attachment to pet dogs and depression in rural older adults. Anthrozoös.

[19] Parslow R.A., Jorm A.F., Christensen H., Rodgers B. (2005). Pet ownership and health in older adults: Findings from a survey of 2551 community-based Australians aged 60–64. Gerontology.

[20] Koivusilta L.K., Ojanlatva A. (2006). To have or not to have a pet for better health?. PLoS ONE.

[21] Walsh F. (2009). Human-animal bonds II: The role of pets in family systems and family therapy. Fam. Process.

[22] Packman W., Field N.P., Carmack B.J., Ronen R. (2011). Continuing bonds and psychosocial adjustment in pet loss. J. Loss Trauma.

[23] Rhodes K.W., Orme J.G., Buehler C. (2001). A comparison of family foster parents who quit, consider quitting, and plan to continue fostering. Soc. Serv. Rev..

[24] Hebert C., Kulkin H.S., McLean M. (2013). Grief and foster parents: How do foster parents feel when a foster child leaves their home?. Adopt. Foster..

[25] Reese L.A. (2018). Strategies for Successful Animal Shelters.

[26] Anderson D.C. (2007). Assessing the Human-Animal Bond. A Compendium of Actual Measures.

[27] Ramírez M.T.G., Quezada Berumen L.D.C., Hernández R.L. (2014). Psychometric properties of the Lexington attachment to pets scale: Mexican version (LAPS-M). Anthrozoös.

[28] McDonald S.E., O’connor K.E., Matijczak A., Tomlinson C.A., Applebaum J.W., Murphy J.L., Zsembik B.A. (2021). Attachment to pets moderates transitions in latent patterns of mental health following the onset of the COVID-19 pandemic: Results of a survey of US adults. Animals.

[29] Nugent W.R., Daugherty L. (2022). A measurement equivalence study of the family bondedness scale: Measurement equivalence between cat and dog owners. Front. Vet. Sci..

[30] Theut S.K., Jordan L., Ross L.A., Deutsch S.I. (1991). Caregiver’s anticipatory grief in dementia: A pilot study. Int. J. Aging Hum. Dev..

[31] Buehler C., Rhodes K.W., Orme J.G., Cuddeback G. (2006). The potential for successful family foster care: Conceptualizing competency domains for foster parents. Child Welf..

[32] Barklam E.B., Felisberti F.M. (2023). Pet ownership and wellbeing during the COVID-19 pandemic: The importance of resilience and attachment to pets. Anthrozoös.

[33] Preston S., Yates K., Moss M. (2012). Does emotional resilience enhance foster placement stability? A qualitative investigation. Int. J. Psychol. Stud..

[34] Smith B.W., deCruz-Dixon N., Schodt K., Torres F., Smith B.W., de Cruz-Dixon N., Schodt K., Torres F. (2023). Brief resilience scale (BRS). Handbook of Assessment in Mindfulness Research.

[35] Reese L.A. (2023). Perception versus policy: Which is more important to animal welfare volunteer satisfaction?. Animals.

[36] Reese L.A. Fostering and the Human-Animal Bond.

[37] Wells D.L., Clements M.A., Elliott L.J., Meehan E.S., Montgomery C.J., Williams G.A. (2022). Quality of the human–animal bond and mental wellbeing during a COVID-19 lockdown. Anthrozoös.

[38] Ackerman R., Watson B., Serpell J., Reinhard C.L., Powell L. (2023). Understanding the motivations of foster caregivers at animal shelters. Animals.

[39] Daily T.A. (2021). Fostering rescued dogs: An exploratory study of the experiences of foster care providers. Hum.-Anim. Interact. Bull..

